# Comparison of culture and culture-free methods for comprehensive identification of mycobacteria: a single-center prospective study

**DOI:** 10.1128/jcm.01128-25

**Published:** 2026-02-27

**Authors:** Kazuki Hashimoto, Kiyoharu Fukushima, Yuki Matsumoto, Haruko Saito, Atsushi Funauchi, Nanako Hamada, June Yamauchi, Tadayoshi Nitta, Daisuke Motooka, Takuro Nii, Takanori Matsuki, Kazuyuki Tsujino, Keisuke Miki, Sho Komukai, Atsushi Kumanogoh, Shota Nakamura, Hiroshi Kida

**Affiliations:** 1 Department of Respiratory Medicine and Clinical Immunology, Graduate School of Medicine, Osaka University 198269 , Suita, Osaka, Japan; 2 Department of Infection Metagenomics, Research Institute for Microbial Diseases, Osaka University 34822, Suita, Osaka, Japan; 3 Department of Clinical Laboratory, National Hospital Organization, Osaka Toneyama Medical Center 38519, Toyonaka, Osaka, Japan; 4 Laboratory of Host Defense, World Premier Institute Immunology Frontier Research Centre (WPI-IFReC), Osaka University, Osaka, Japan; 5 Department of Respiratory Medicine, National Hospital Organisation, Osaka Toneyama Medical Centre, Toyonaka, Osaka, Japan; 6 Department of Health Data Science, Tokyo Medical University 13112, Shinjuku, Tokyo, Japan; 7 Integrated Frontier Research for Medical Science Division, Institute for Open and Transdisciplinary Research Initiatives, Osaka University, Suita, Japan; 8 Centre for Infectious Disease Education and Research, Osaka University 13013, Suita, Japan; University of Western Australia, Perth, Australia

**Keywords:** nontuberculous mycobacteria, tuberculosis, target capture sequencing, multi-locus sequence typing

## Abstract

The genus *Mycobacterium*, including *Mycobacterium tuberculosis* and over 200 nontuberculous mycobacteria (NTM), shows wide variability in clinical outcomes and drug susceptibility. Although culture-based identification remains the gold standard, slow mycobacterial growth delays diagnosis and treatment. In this study, we evaluated a novel culture-free method for subspecies-level identification directly from sputum. In this single-center prospective cohort study at Osaka Toneyama Medical Center, we analyzed 125 sputum samples from 115 patients with NTM pulmonary disease and 10 with non-NTM respiratory conditions. Samples were decontaminated using N-acetyl-L-cysteine–sodium hydroxide (NALC-NaOH) or succinic acid. We compared the reference culture method (mycobacterial culture plus whole-genome sequencing) and a culture-free direct target capture sequencing method. Core genome multi-locus sequence typing identified subspecies in both workflows, covering 186 mycobacterial species, including *M. tuberculosis*. The 115 NTM cohort specimens yielded 57 smear-positive and 93 culture-positive results. The identified subspecies included 48 *Mycobacterium avium* subsp. *hominissuis*, 22 *Mycobacterium intracellulare* subsp. *intracellulare*, 5 subsp. *chimaera*, 7 *Mycobacterium abscessus* subsp. *abscessus*, 5 subsp. *massiliense*, 1 *M. tuberculosis*, and 5 other NTM species. The culture-free method showed a high identification rate for smear-positive specimens (75.4%) but a low identification rate for smear-negative specimens (13.9%). NALC-NaOH pretreatment resulted in higher accuracy (90.5%) than did succinic acid pretreatment (66.7%). Thus, our culture-free subspecies-level identification method achieved high accuracy, especially in alkaline-treated smear-positive sputum samples, achieving rates above 90%. This method is recommended in clinical practice for patients who require rapid diagnosis and timely initiation of appropriate treatment, bypassing time-consuming culture steps.

**IMPORTANCE:**

Accurate identification of *Mycobacterium* species and subspecies is crucial for effective treatment, as drug susceptibility and clinical outcomes vary significantly among them. However, conventional diagnosis relies on culture-based methods that can take several weeks, critically delaying appropriate therapy. This study validates a novel culture-free method using target capture sequencing for the comprehensive, subspecies-level identification of over 186 mycobacterial species directly from sputum specimens. Our findings revealed the high accuracy of this approach for smear-positive specimens, especially with alkaline pretreatment. This rapid method is applicable in clinical settings and enables timely and precise treatment decisions, greatly benefiting patients who require urgent intervention.

## INTRODUCTION

Nontuberculous mycobacterial pulmonary disease (NTM-PD) is increasingly prevalent worldwide and is a significant global health challenge (1). Notably, the prevalence of NTM-PD in Japan has markedly increased over the past few decades, surpassing that of tuberculosis (2, 3). NTM encompasses over 200 species, of which 140 are pathogenic, and the remaining 60 are considered potentially pathogenic (4–6). Accurate and comprehensive subspecies-level identification is crucial, as drug susceptibility profiles and clinical outcomes substantially vary not only between species but also among subspecies of major pathogenic species, such as *Mycobacterium abscessus* and *Mycobacterium intracellulare* (7, 8).

Conventional diagnostic methods for mycobacteria have some limitations. Rapid molecular assays such as polymerase chain reaction (PCR) and transcription–reverse transcription concerted reaction (TRC) often identify only a limited range of species (9), while more comprehensive techniques such as matrix-assisted laser desorption/ionization time-of-flight mass spectrometry frequently lack subspecies resolution (10). Therefore, when clinically important species such as *M. abscessus* are identified, additional tests such as immunochromatography or multiplex PCR are required to determine the subspecies (11, 12). This necessitates sequential testing, leading to diagnostic complexity and delays. These limitations highlight the need for a rapid and comprehensive method for subspecies-level identification. Advances in next-generation sequencing have enabled rapid and comprehensive genomic analysis, offering a potential solution to these challenges. We developed mycobacteria growth indicator tube sequencing (MGIT-Seq), which uses core genome multi-locus sequence typing (cgMLST) based on whole-genome sequencing that enables comprehensive species- and subspecies-level identification from MGIT culture within 3 days. MGIT-Seq yielded 99.1% accuracy in species-level identification and identified 84.5% of the isolates at the subspecies level (13). However, MGIT-Seq still involves a culture step. Mycobacteria grow slowly, often requiring 1–8 weeks, which hinders timely diagnosis and treatment.

We recently developed NALC-Seq, a novel method for the comprehensive culture-free identification of mycobacteria. This method uses target capture sequencing designed for mycobacteria and enables direct identification from sputum pretreated with N-acetyl-L-cysteine–NaOH (NALC-NaOH), bypassing the need for culture (14). However, its clinical performance has not been validated, including culture-negative specimens and non-mycobacterial cases. NALC-NaOH and succinic acid are commonly used for sputum decontamination in clinical practice; however, the optimal pretreatment strategy for direct molecular analysis remains unclear.

To address these gaps, we aimed to validate the diagnostic accuracy of the culture-free method for subspecies-level identification of mycobacteria in a clinical cohort and determine the optimal sputum pretreatment by comparing the effects of NALC-NaOH and succinic acid.

## MATERIALS AND METHODS

### Study design and patients

In this single-center, prospective cohort study, we recruited 115 patients diagnosed with NTM-PD according to American Thoracic Society/European Respiratory Society/European Society of Clinical Microbiology and Infectious Diseases/Infectious Diseases Society of America (ATS/ERS/ESCMID/IDSA) diagnostic criteria at Osaka Toneyama Medical Center (15). The exclusion criterion was age <18 years. Sputum specimens were collected from these patients between March 2023 and July 2024. If a patient provided multiple specimens, we included only the most recent specimen in the analysis. These 115 patients were categorized into the NTM cohort group.

In December 2024, we collected sputum specimens from 10 patients who visited our hospital for conditions other than mycobacterial infections, and these patients were categorized as the non-NTM control group. Patients with chest computed tomography (CT) findings suggestive of NTM-PD (such as cavities, bronchiectasis, or nodular opacities) were excluded from this control group.

**Fig 1:**
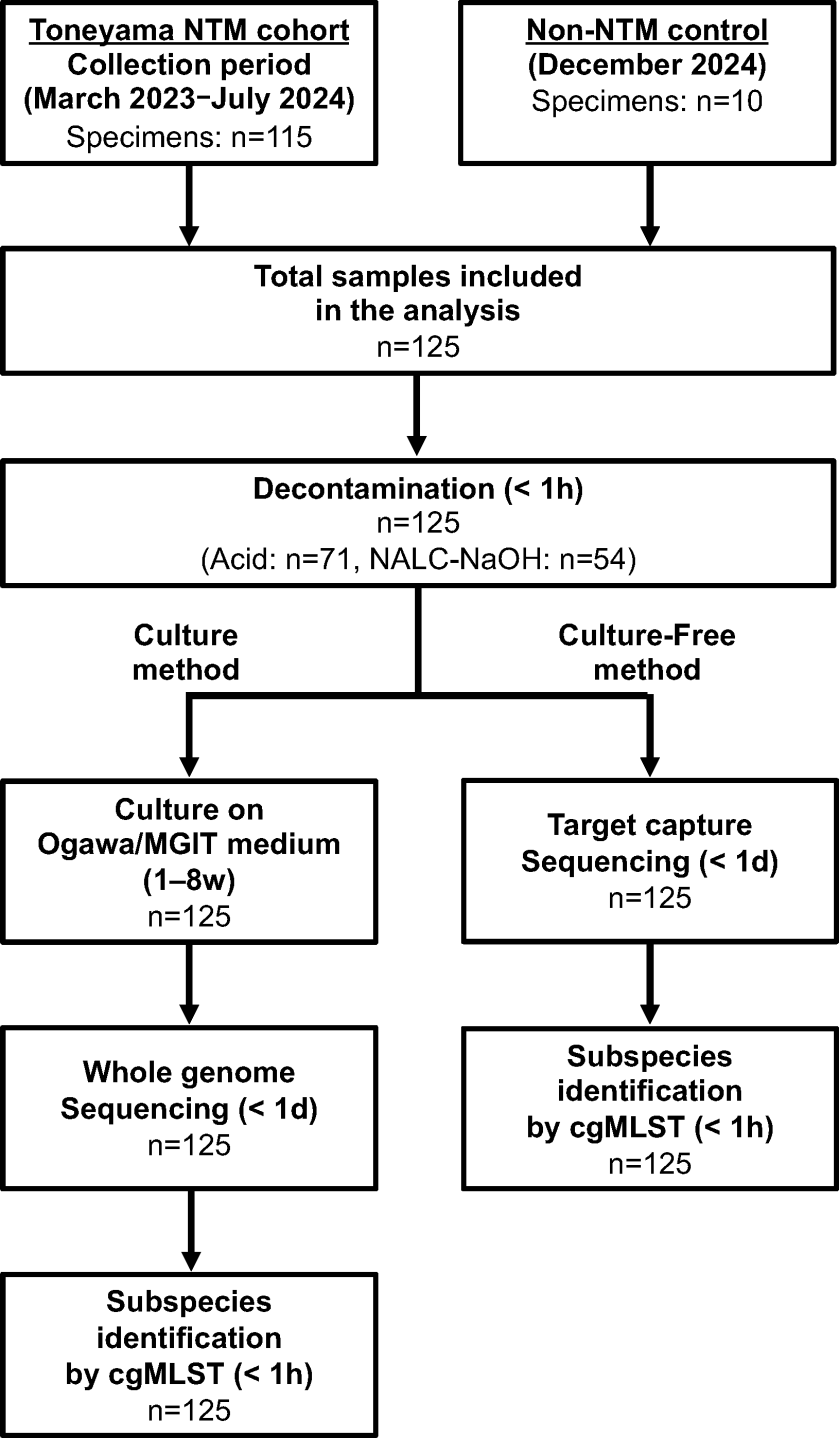
Study design NTM-PD, nontuberculous mycobacterial pulmonary disease; SAP, semi-alkaline protease; NALC-NaOH, N-acetyl-L-cysteine-NaOH; MGIT, mycobacterial growth indicator tube; cgMLST, core genome multi-locus sequence typing.

Overall, 125 sputum specimens from 125 patients were analyzed (Fig. 1).

### Baseline characteristics

Patients’ baseline characteristics included age, sex, body mass index, smoking history, erythrocyte sedimentation rate (ESR), anti-glycopeptidolipid (GPL)-core immunoglobulin A antibody levels, the presence of cavities on CT, and the use of multidrug therapy for NTM at the sampling time. The results of acid-fast bacilli (AFB) tests of sputum samples were also recorded, including smear results (negative, ±, 1+, 2+, or 3+) (16). In addition, the culture results (positive or negative) and the pretreatment method (NALC-NaOH or succinic acid) were recorded. ESR was defined as elevated if it exceeded 15 mm/h in men or 20 mm/h in women, according to previously reported criteria (17). Anti-GPL-core IgA antibody levels ≥0.7 U/mL were interpreted as positive, as described in a previous study (18).

### Sputum pretreatment

Sputum specimens were processed as follows: For mucolysis, each specimen was mixed with approximately three times its volume of semi-alkaline protease solution and incubated at room temperature for 20 min with gentle agitation. The mixture was centrifuged (3,000 ×*g*, 20 min) using a refrigerated centrifuge. The pellet was then resuspended in sterile phosphate-buffered saline (PBS) to 0.5 mL.

Decontamination was performed using either NALC-NaOH or succinic acid, depending on the physician’s choice of subsequent culture medium: NALC-NaOH for MGIT medium and succinic acid for Ogawa medium. NALC-NaOH was applied using the BD MycoPre kit (Becton, Dickinson and Company, New Jersey, USA), and succinic acid was added using Sputamentsol (Kyokuto Pharmaceutical, Tokyo, Japan), both according to the manufacturers’ instructions.

After decontamination (irrespective of the method used), the specimens were neutralized with 0.067 M PBS (pH 6.8), centrifuged (3,000 ×*g*, 20 min), and washed once with PBS. Then, the pellet was resuspended in 1 mL PBS.

### Study workflow

Following pretreatment, each specimen was analyzed for mycobacterial identification using the following workflows: (1) a culture method, in which pretreated sputum was cultured, followed by whole-genome sequencing of the cultured isolates; and (2) a culture-free method, in which pretreated sputum was directly analyzed by target capture sequencing using custom-designed RNA probes specific to the mycobacterial genome. cgMLST identified the subspecies across both workflows. The performance of the culture-free method was evaluated using the culture method as the reference standard. K.H. and Y.M. were blinded to the clinical data. K.H. performed the culture-free method, while Y.M. performed the culture method.

### Culture method

Sputum specimens pretreated with NALC-NaOH were cultured in MGIT medium (Becton Dickinson, Franklin Lakes, USA), while those pretreated with succinic acid were cultured in Ogawa medium (Kyokuto Pharmaceutical, Tokyo, Japan). Once culture positivity was confirmed, genomic deoxyribonucleic acid (DNA) was extracted using the DNeasy PowerSoil Pro Kit (QIAGEN, Baarn, Netherlands). Sequencing libraries were prepared with the Ligation Sequencing Kit V14 (SQK-LSK114) and Native Barcoding Kit 96 V14 (SQK-NBD114.96) from Oxford Nanopore Technologies (Oxford, UK). Whole-genome sequencing was conducted on the P2 Solo platform (PRO-SEQ002) using R10.4.1 flow cells (FLO-PRO114M).

### Culture-free method

Following pretreatment, genomic DNA was extracted through mechanical disruption using bead beating with 0.1-mm glass beads (MN Bead Tubes Type B, Takara Bio, Shiga, Japan), followed by centrifugation at 13,000 rpm for 5 min to remove debris. The supernatant containing genomic DNA was heat-treated at 95°C for 5 min to deactivate contaminants and stabilize DNA. The extracted DNA was quantified, and approximately 150 ng was used for library preparation using the SureSelect Low Input Target Enrichment System (Agilent Technologies, Santa Clara, CA, USA). Target enrichment was achieved with proprietary RNA probes designed by our research team using SureDesign software to cover the 184 loci used in cgMLST. These 184 conserved loci include reference housekeeping genes as well as genes related to ribosomal proteins, ribosomal RNA, antibiotic resistance, and pathogenicity (Table S1). Paired-end sequencing with 150 base pair reads in both directions was performed on DNBSEQ-G400RS (MGI Tech Co., Ltd., Shenzhen, China).

### cgMLST analysis

Subspecies-level identification for the culture and culture-free methods was carried out using cgMLST (19). The genetic profiles identified from the 184 conserved loci described above were compared with those in our custom reference database. This database contains profiles for 186 *Mycobacterium* species (comprising 389 subspecies), including *Mycobacterium tuberculosis* complex (Table S2). Subspecies were identified on the basis of the profile with the highest score.

### Statistical analysis

Continuous variables are summarized as medians with interquartile ranges, while categorical variables are presented as frequencies and percentages. Continuous and categorical variables were compared between the two groups using the Wilcoxon rank-sum and Fisher’s exact tests, respectively.

## RESULTS

### Study participants

The baseline characteristics of the 125 patients are summarized in Table 1. Of those in the NTM cohort group (*n* = 115), 93 (80.9%) patients were AFB culture-positive, and 57 (49.6%) were smear-positive. The non-NTM control group (*n* = 10) included patients with lung cancer (*n* = 6), abnormal chest shadows (*n* = 2), Mycoplasma pneumonia (*n* = 1), and pneumoconiosis (*n* = 1). All control specimens were AFB smear- and culture-negative.

**TABLE 1:** Clinical characteristics of the study population^*a*^^,^^*b*^

	Total	NTM cohort group	non-NTM control group
	*n* = 125	*n* = 115	*n* = 10
Sex, female	92 (73.6%)	88 (76.5%)	4 (40.0%)
Age, years	75.1 (66.4–82.3)	74.5 (66.3–82.3)	79.6 (71.1–82.4)
Body mass index, kg/m^2^	19.0 (17.2–20.9)	18.8 (17.1–20.5)	22.3 (19.8–23.4)
Cavity	40 (32.0%)	40 (34.8%)	0 (0.0%)
Elevated ESR (*n* = 25)	13/25 (52.0%)	13/25 (52.0%)	N.A.
Anti-GPL-core IgA antibody-positive (*n* = 62)	51/62 (82.3%)	51/62 (82.3%)	N.A.
Smoking history	43 (34.4%)	36 (31.3%)	7 (70.0%)
Multidrug therapy for mycobacteria at sampling	44 (35.2%)	44 (38.2%)	0 (0.0%)
AFB smear-positive	57 (45.6%)	57 (49.6%)	0 (0.0%)
AFB culture-positive	93 (74.4%)	93 (80.9%)	0 (0.0%)
Pretreatment method			
NALC-NaOH	54 (43.2%)	45 (39.1%)	9 (90.0%)
Succinic acid	71 (56.8%)	70 (60.9%)	1 (10.0%)

^*a*^ NTM, nontuberculous mycobacteria; ESR, erythrocyte sedimentation rate; GPL, glycopeptidolipid; IgA, immunoglobulin A; AFB, acid-fast bacilli; NALC-NaOH, N-acetyl-L-cysteine-sodium hydroxide; N.A., not applicable.

^*b*^ Data are presented as number (%) or median (interquartile range).

### Mycobacterial Identification by culture method

For the 93 culture-positive specimens from the NTM cohort, the culture method identified a diverse spectrum of mycobacteria, as detailed in Table 2. These included *Mycobacterium avium* subsp. *hominissuis* in 48 cases (51.6%); *M. intracellulare* subsp. *intracellulare* in 22 (23.7%); *M. intracellulare* subsp. *chimaera* in 5 (5.4%); *M. abscessus* subsp. *abscessus* in 7 (7.5%); *M. abscessus* subsp. *massiliense* in 5 (5.4%); and *M. tuberculosis*, *Mycobacterium kansasii*, *Mycobacterium paragordonae*, *Mycobacterium mucogenicum*, *Mycobacterium simiae*, and unclassified mycobacterium in 1 case each. The unclassified mycobacterium was subsequently identified as *Tsukamurella carboxydivorans*, an oral commensal, on the basis of an average nucleotide identity of 99.91%. No differences were observed in species distribution, including rapidly growing mycobacteria, between the pretreatment methods.

**TABLE 2:** Identification results by culture methods for the 93 culture-positive patients^*a*^

Species identified by cgMLST	Total	Pretreatment	
	*n* = 93	NALC-NaOH *n* = 39	Acid *n* = 54
*M. avium* subsp. *hominissuis*	48	21	27
*M. intracellulare* subsp. *intracellulare*	22	5	17
*M. intracellulare* subsp. *chimaera*	5	4	1
*M. abscessus* subsp. *abscessus*	7	5	2
*M. abscessus* subsp. *massiliense*	5	0	5
*M. tuberculosis*	1	1	0
*M. kansasii*	1	1	0
*M. paragordonae*	1	0	1
*M. mucogenicum*	1	1	0
*M. simiae*	1	0	1
Unclassified mycobacteria^*b*^	1	1	0

^*a*^ cgMLST, core genome multi-locus sequence typing; *M. avium*, *Mycobacterium avium; M. intracellulare*, *Mycobacterium intracellulare; M. abscessus*, *Mycobacterium abscessus; M. tuberculosis*, *Mycobacterium tuberculosis; M. kansasii*, *Mycobacterium kansasii; M. paragordonae*, *Mycobacterium paragordonae; M. mucogenicum*, *Mycobacterium mucogenicum; M. simiae*, *Mycobacterium simiae*; subsp., subspecies.

^*b*^ Subsequently identified as *Tsukamurella carboxydivorans* by average nucleotide identity analysis.

### Diagnostic performance of the culture-free method

**Fig 2:**
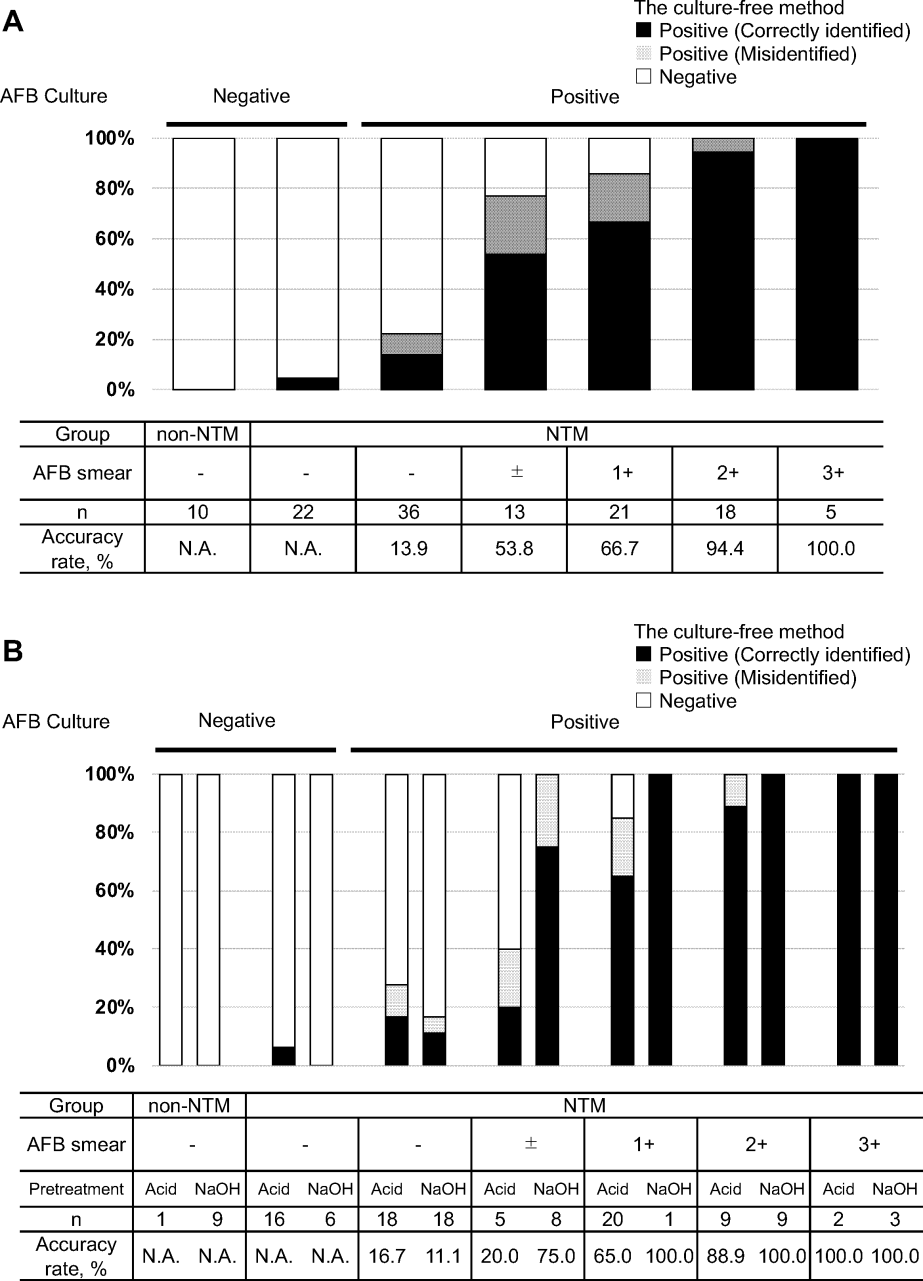
Diagnostic performance of the culture-free method, compared with the culture method (**A**) Accuracy stratified by smear status, culture status, and patient group. (**B**) Accuracy by pretreatment protocol (acid *vs*. NALC-NaOH). NTM, nontuberculous mycobacteria; AFB, acid-fast bacilli; NALC-NaOH, N-acetyl-L-cysteine–sodium hydroxide.

Figure 2 illustrates the diagnostic performance of the culture-free method, compared with that of the culture method. All non-NTM control specimens (*n* = 10) tested negative with the culture-free method.

In the NTM cohort group, the diagnostic performance varied according to the smear and culture status. The method yielded negative results for 21 out of the 22 smear-negative and culture-negative specimens (95.5%), with *M. intracellulare* detected in 1 case. For smear-negative, culture-positive specimens (*n* = 36), the performance was limited. The method correctly identified the subspecies in 5 cases (13.9%), including one with *M. tuberculosis*. Misidentification as other NTM was observed for 3 cases (8.3%), while negative results were achieved for 28 cases (77.8%). The accuracy of NALC-NaOH pretreatment (11.1%, 2/18) and acid pretreatment (16.7%, 3/18) was comparable in smear-negative, culture-positive specimens (*P* = 1.000).

In contrast, the culture-free method showed better performance in smear-positive, culture-positive specimens (*n* = 57), correctly identifying the subspecies in 43 cases (75.4%), misidentifying in 8 cases (14.0%), and yielding negative results in 6 cases (10.5%). Accuracy increased with smear grade (53.8% for ±, 66.7% for 1+, 94.4% for 2+, and 100% for 3+), and it was numerically higher with NALC-NaOH pretreatment (90.5%, 19/21) than with acid pretreatment (66.7%, 24/36) (*P* = 0.058) in smear-positive, culture-positive specimens.

Detailed species-specific performance is summarized in Table 3. Overall, in each species, high specificities were observed in both AFB smear negative and positive specimens, sensitivities were high in AFB smear positive specimens and low in AFB smear negative specimens. *M. avium*, *M. intracellulare*, and *M. abscessus* were accurately identified at the subspecies level with high positive predictive values, and no major differences in accuracy were observed across species. Although the number of cases was small, *M. tuberculosis* and *M. kansasii* were accurately identified. Further investigation is needed into rarer species, such as *M. paragorodonae*, *M. gorodonae*, *M. mucogenicum,* and *M. simae.*

**TABLE 3:** Species-specific diagnostic performance of the culture-free method compared with that of the culture method^*a*^^,^^*b*^^,^^*c*^

AFB smear	Species	Sensitivity	Specificity	PPV	NPV
−	*M. avium* subsp. *hominissuis*	9.1% (2/22)	100.0% (46/46)	100.0% (2/2)	69.7% (46/66)
	*M. intracellulare* subsp. *intracellulare*	20.0% (1/5)	98.4% (62/63)	50.0% (1/2)	93.9% (62/66)
	*M. intracellulare* subsp. *chimaera*	0.0% (0/2)	100.0% (66/66)	N.A. (0/0)	100.0% (68/68)
	*M. abscessus* subsp. *abscessus*	33.3% (1/3)	98.5% (64/65)	50.0% (1/2)	97.0% (64/66)
	*M. abscessus* subsp. *massiliense*	0.0% (0/1)	100.0% (67/67)	N.A. (0/0)	98.5% (67/68)
	*M. tuberculosis*	100.0% (1/1)	100.0% (67/67)	100.0% (1/1)	100.0% (67/67)
	*M. paragordonae*	0.0% (0/1)	100.0% (67/67)	N.A. (0/0)	98.5% (67/68)
+	*M. avium* subsp. *hominissuis*	73.1% (19/26)	93.5% (29/31)	90.5% (19/21)	80.6% (29/36)
	*M. intracellulare* subsp. *intracellulare*	82.4% (14/17)	97.5% (39/40)	93.3% (14/15)	92.9% (39/42)
	*M. intracellulare* subsp. *chimaera*	66.7% (2/3)	100.0% (54/54)	100.0% (2/2)	98.2% (54/55)
	*M. abscessus* subsp. *abscessus*	100.0% (4/4)	100.0% (53/53)	100.0% (4/4)	100.0% (53/53)
	*M. abscessus* subsp. *massiliense*	75.0% (3/4)	100.0% (53/53)	100.0% (3/3)	98.1% (53/54)
	*M. kansasii*	100.0% (1/1)	100.0% (56/56)	100.0% (1/1)	100.0% (56/56)
	*M. mucogenicum*	0.0% (0/1)	100.0% (56/56)	N.A. (0/0)	98.2% (56/57)
	*M. simiae*	0.0% (0/1)	100.0% (56/56)	N.A. (0/0)	98.2% (56/57)

^*a*^ AFB: acid-fast bacilli; PPV: positive predictive value; NPV: negative predictive value; −: negative; +: positive; *M. avium: Mycobacterium avium; M. intracellulare: Mycobacterium intracellulare; M. abscessus: Mycobacterium abscessus; M. tuberculosis: Mycobacterium tuberculosis; M. paragordonae: Mycobacterium paragordonae; M. kansasii: Mycobacterium kansasii; M. mucogenicum: Mycobacterium mucogenicum; M. simiae: Mycobacterium simiae*; subsp.: subspecies; N.A.: not applicable.

^*b*^ Data are presented as number (%).

^*c*^ Performance metrics were calculated for each NTM species using 2 × 2 contingency tables comparing the results of the culture-free and culture methods.

## DISCUSSION

In this prospective cohort study, we demonstrated that a culture-free method using targeted capture sequencing facilitated comprehensive subspecies-level mycobacterial identification directly from sputum. The accuracy of the method showed a clear dependence on bacterial load, achieving 75.4% in smear-positive specimens compared with 13.9% in smear-negative specimens. With regard to pretreatment, NALC-NaOH pretreatment yielded higher accuracy than did succinic acid pretreatment (90.5% vs 66.7%). By eliminating culture steps, this method enables same-day subspecies-level identification and can facilitate early clinical decisions.

 Direct identification methods for mycobacteria differ fundamentally in terms of the diagnostic approach, which determines their species coverage, turnaround time, and subspecies-level resolution. Targeted molecular assays such as multiplex PCR and line probe assays provide results within hours and show high species-level accuracy in smear-positive specimens (80.0% and 91.8%, respectively) (9, 20). However, they can detect only a limited set of predefined species, and subspecies-level discrimination is largely restricted to *M. abscessus*. Recent panel-based sequencing and hybridization assays have shown increased diagnostic coverage. Deeplex Myc-TB identifies 146 species with 84.2% accuracy, while Mycopanel identifies 30 species with 83.1% accuracy (21, 22). Because both depend on predefined genetic targets, subspecies-level discrimination remains limited to a small subset of clinically important taxa. Our target capture sequencing system addresses these limitations by comprehensively enriching mycobacterial DNA directly from sputum and generating more informative sequence data than do existing panel-based assays. Leveraging a reference database comprising 186 species and 389 subspecies, it enables comprehensive species- and subspecies-level identification within 24 h. Although many NTM species exist, our reference database already includes most clinically important taxa, such as *M. avium*, *M. intracellulare*, *M. abscessus*, *Mycobacterium xenopi*, *Mycobacterium fortuitum*, and *Mycobacterium gordonae*. Because these major pathogens are commonly encountered across different regions and healthcare settings, the current panel is expected to have broad applicability. Validation using additional, less common species will further extend its utility. Probe-based hybridization assays are generally resource-intensive, and the estimated reagent cost for our method is approximately 98.6 USD per sample, as shown in Table S3. 

The diagnostic performance of the culture-free method increased in a stepwise manner with higher smear grades. This trend is consistent with those shown by other direct molecular methods (9, 23–25), reinforcing the principle that high mycobacterial load is a major factor for successful direct identification. Notably, international guidelines often consider smear-positive results as a significant criterion for initiating treatment for certain mycobacterial infections (15, 26). Accordingly, we recommend applying the culture-free method primarily to smear-positive specimens, especially in cases requiring prompt treatment, such as those with fibrocavitary disease, extensive nodular–bronchiectatic lesions, or hemoptysis.

The success of direct mycobacterial sequencing critically depends on sample pretreatment. NALC-NaOH decontamination is known to potentially degrade DNA, particularly that of rapidly growing mycobacteria (27), and reportedly reduces the success of direct whole-genome sequencing for *M. tuberculosis* (28). In our study, however, NALC-NaOH pretreatment resulted in greater success of sequencing than did acid pretreatment, probably because of the weaker decontamination effect of acid treatment that allowed background DNA to persist, which may have caused nonspecific cross-reactivity with our mycobacterial-specific probes. Therefore, for this method, NALC-NaOH decontamination is recommended, as the benefit of reducing nonspecific hybridization outweighs the potential risk of DNA degradation. Beyond pretreatment optimization, decontamination-free workflows also warrant evaluation, as recent studies have shown that capture probes can selectively hybridize even in the presence of background DNA (25).

A further challenge with the culture-free method is the low sensitivity (13.9%) observed in smear-negative, culture-positive specimens. Because of the low sensitivity, clinicians hesitate to use this method for smear-negative patients, who are commonly encountered in routine clinical practice (29). To address this critical issue, future approaches should focus on maximal bacterial DNA recovery, improved capture probe specificity, and host DNA depletion (28). Because low-load specimens contain very little mycobacterial DNA, maximizing recovery and minimizing even minor loss during extraction are crucial. Although bead-beating and boiling are robust for lysis, further optimization of extraction and concentration is necessary to improve detection sensitivity in scanty and smear-negative specimens (28).

Our method represents a major step toward the realization of rapid and comprehensive sequencing directly in clinical settings. First, the workflow is designed to be performed without the need for expertise in bioinformatics. The analysis pipeline used for processing sputum samples in this study, which included base calling, mapping against the reference database, and score-based filtering, is automated. The identified species can be reviewed on our website (14). Second, this direct identification approach offers significant platform flexibility. The culture-free method in this study utilized the DNBSEQ platform, with consideration of throughput and cost-efficiency for processing multiple samples. However, this approach is also adaptable to MinION portable sequencers, which are not only suitable for use in clinical microbiology laboratories but also capable of simultaneous sequencing and analysis. In the pilot study evaluating the applicability of MinION, the turnaround time was shortened to approximately 19 h (14).

This study also had some limitations. First, because it was a single-center investigation, our findings may not be generalizable to diverse clinical settings or patient populations. Second, insufficient residual sputum volume precluded direct comparative analysis with conventional PCR and TRC assays, preventing assessment of diagnostic performance against current rapid tests. Third, this method was optimized to identify only the most dominant species, and it has not been validated for detecting mixed infections. In regions where tuberculosis is highly prevalent, mixed infections involving NTM could lead to missed tuberculosis diagnoses if *M. tuberculosis* is not dominant. Therefore, implementation in such settings will require further optimization of probe design and the analytical pipeline, as well as validation using confirmed *M. tuberculosis*–NTM and NTM–NTM mixed infection specimens.

In conclusion, our culture-free method using NALC-NaOH pretreatment enabled subspecies-level identification of mycobacteria directly from smear-positive sputum with over 90% accuracy. By covering a broad range of mycobacterial species and eliminating the need for culture, this approach shortens the diagnostic timeline and offers practical clinical utility for early therapeutic decision-making.

## Data Availability

The datasets supporting the conclusions of this study are included in this article. The datasets generated and analyzed here are available from the corresponding author upon reasonable request. The raw sequencing data supporting this study’s findings have been deposited in the DDBJ and NCBI under BioProject accession numbers PRJDB12894, PRJNA1273648, and PRJNA1280714 (Table S4).

## SUPPLEMENTAL MATERIAL

Table S1 jcm.01128-25-s0001.xlsx List of the 184 conserved loci.

Table S2 jcm.01128-25-s0002.xlsx RefSeq list for cgMLST.

Table S3 jcm.01128-25-s0003.docx Costs for Culture-free method.

Table S4 jcm.01128-25-s0004.docx Data mapping table.

ASM does not own the copyrights to Supplemental Material that may be linked to, or accessed through, an article. The authors have granted ASM a non-exclusive, world-wide license to publish the Supplemental Material files. Please contact the corresponding author directly for reuse.
