# Supplementary material for: Comparison of culture and culture-free methods for comprehensive identification of mycobacteria: a single-center prospective study
Source: J Clin Microbiol. 2026 Feb 27;64(4):e01128-25. doi: 10.1128/jcm.01128-25 (PMC13059708; doi:10.1128/jcm.01128-25)
Supplement: Table S3 — Costs for Culture-free method. [file jcm.01128-25-s0003.docx]

Table S3. Costs for Culture-free method

|  | Total cost | Samples covered | Cost per sample |
| --- | --- | --- | --- |
| DNA extraction | $9.9 | 1 | $9.9 |
| Library preparation |  |  |  |
| General consumables | $33.0 | 1 | $33.0 |
| SURESELECT XT HS2 Reagent Kit with Index Primer Pairs (G9983A) | $3448.7 | 96 | $35.9 |
| SureSelect Enzymatic Fragmentation Kit (5191-6764) | $592.7 | 96 | $6.2 |
| Capture probes (SureSelect PostPool Custom Tier2, S3379912) | $8000.0 | 960 | $8.3 |
| Sequencing run (DNBSEQ-G400RS) | $660.0 | 125 | $5.3 |
|  |  |  | $98.6 |

The costs were calculated based on the list prices in Japan as of November 2025.
