# Supplementary material for: Comparison of culture and culture-free methods for comprehensive identification of mycobacteria: a single-center prospective study
Source: J Clin Microbiol. 2026 Feb 27;64(4):e01128-25. doi: 10.1128/jcm.01128-25 (PMC13059708; doi:10.1128/jcm.01128-25)
Supplement: Table S4 — Data mapping table. [file jcm.01128-25-s0004.docx]

**Table S4. Data mapping table**

| No. | Specimen | BioProject Accession | Run Accession |
| --- | --- | --- | --- |
| 1 | Sputum | PRJNA1280714 | SRR34116600 |
|  | Culture | PRJDB19189 | DRR629252 |
| 2 | Sputum | PRJNA1280714 | SRR34116599 |
|  | Culture | PRJDB12894 | DRR629253 |
| 3 | Sputum | PRJNA1280714 | SRR34116531 |
|  | Culture | Negative | Negative |
| 4 | Sputum | PRJNA1280714 | SRR34116520 |
|  | Culture | Negative | Negative |
| 5 | Sputum | PRJNA1280714 | SRR34116509 |
|  | Culture | PRJDB19189 | DRR629259 |
| 6 | Sputum | PRJNA1280714 | SRR34116594 |
|  | Culture | Negative | Negative |
| 7 | Sputum | PRJNA1280714 | SRR34116583 |
|  | Culture | PRJDB12894 | DRR629260 |
| 8 | Sputum | PRJNA1280714 | SRR34116572 |
|  | Culture | PRJDB19189 | DRR629328 |
| 9 | Sputum | PRJNA1280714 | SRR34116497 |
|  | Culture | PRJDB19189 | DRR629329 |
| 10 | Sputum | PRJNA1280714 | SRR34116486 |
|  | Culture | PRJDB19189 | DRR629261 |
| 11 | Sputum | PRJNA1280714 | SRR34116598 |
|  | Culture | PRJDB19189 | DRR629330 |
| 12 | Sputum | PRJNA1280714 | SRR34116555 |
|  | Culture | PRJDB19189 | DRR629332 |
| 13 | Sputum | PRJNA1280714 | SRR34116544 |
|  | Culture | PRJDB19189 | DRR629334 |
| 14 | Sputum | PRJNA1280714 | SRR34116538 |
|  | Culture | PRJDB19189 | DRR629335 |
| 15 | Sputum | PRJNA1280714 | SRR34116537 |
|  | Culture | PRJDB19189 | DRR629337 |
| 16 | Sputum | PRJNA1280714 | SRR34116536 |
|  | Culture | PRJDB19189 | DRR629338 |
| 17 | Sputum | PRJNA1280714 | SRR34116535 |
|  | Culture | PRJDB19189 | DRR629339 |
| 18 | Sputum | PRJNA1280714 | SRR34116534 |
|  | Culture | PRJDB19189 | DRR629340 |
| 19 | Sputum | PRJNA1280714 | SRR34116533 |
|  | Culture | PRJDB19189 | DRR629341 |
| 20 | Sputum | PRJNA1280714 | SRR34116532 |
|  | Culture | PRJNA1273648 | SRR33884416 |
| 21 | Sputum | PRJNA1280714 | SRR34116530 |
|  | Culture | PRJDB12894 | DRR629266 |
| 22 | Sputum | PRJNA1280714 | SRR34116529 |
|  | Culture | PRJDB12894 | DRR629267 |
| 23 | Sputum | PRJNA1280714 | SRR34116528 |
|  | Culture | PRJNA1273648 | SRR34136514 |
| 24 | Sputum | PRJNA1280714 | SRR34116527 |
|  | Culture | Negative | Negative |
| 25 | Sputum | PRJNA1280714 | SRR34116526 |
|  | Culture | PRJDB12894 | DRR629268 |
| 26 | Sputum | PRJNA1280714 | SRR34116525 |
|  | Culture | PRJNA1273648 | SRR33884415 |
| 27 | Sputum | PRJNA1280714 | SRR34116524 |
|  | Culture | Negative | Negative |
| 28 | Sputum | PRJNA1280714 | SRR34116523 |
|  | Culture | Negative | Negative |
| 29 | Sputum | PRJNA1280714 | SRR34116522 |
|  | Culture | PRJDB12894 | DRR629306 |
| 30 | Sputum | PRJNA1280714 | SRR34116521 |
|  | Culture | PRJDB12894 | DRR629307 |
| 31 | Sputum | PRJNA1280714 | SRR34116519 |
|  | Culture | PRJDB12894 | DRR629309 |
| 32 | Sputum | PRJNA1280714 | SRR34116518 |
|  | Culture | PRJNA1273648 | SRR33884404 |
| 33 | Sputum | PRJNA1280714 | SRR34116517 |
|  | Culture | PRJNA1273648 | SRR33884393 |
| 34 | Sputum | PRJNA1280714 | SRR34116516 |
|  | Culture | PRJDB12894 | DRR629310 |
| 35 | Sputum | PRJNA1280714 | SRR34116515 |
|  | Culture | PRJNA1273648 | SRR33884383 |
| 36 | Sputum | PRJNA1280714 | SRR34116514 |
|  | Culture | PRJDB12894 | DRR629311 |
| 37 | Sputum | PRJNA1280714 | SRR34116513 |
|  | Culture | PRJDB12894 | DRR629206 |
| 38 | Sputum | PRJNA1280714 | SRR34116512 |
|  | Culture | Negative | Negative |
| 39 | Sputum | PRJNA1280714 | SRR34116511 |
|  | Culture | PRJDB12894 | DRR629313 |
| 40 | Sputum | PRJNA1280714 | SRR34116510 |
|  | Culture | PRJNA1273648 | SRR33884382 |
| 41 | Sputum | PRJNA1280714 | SRR34116508 |
|  | Culture | PRJDB12894 | DRR629314 |
| 42 | Sputum | PRJNA1280714 | SRR34116507 |
|  | Culture | PRJDB12894 | DRR629316 |
| 43 | Sputum | PRJNA1280714 | SRR34116506 |
|  | Culture | Negative | Negative |
| 44 | Sputum | PRJNA1280714 | SRR34116505 |
|  | Culture | PRJDB12894 | DRR629317 |
| 45 | Sputum | PRJNA1280714 | SRR34116504 |
|  | Culture | PRJDB12894 | DRR629318 |
| 46 | Sputum | PRJNA1280714 | SRR34116503 |
|  | Culture | PRJDB12894 | DRR629319 |
| 47 | Sputum | PRJNA1280714 | SRR34116502 |
|  | Culture | PRJDB12894 | DRR629320 |
| 48 | Sputum | PRJNA1280714 | SRR34116501 |
|  | Culture | PRJDB12894 | DRR629321 |
| 49 | Sputum | PRJNA1280714 | SRR34116596 |
|  | Culture | PRJDB12894 | DRR629322 |
| 50 | Sputum | PRJNA1280714 | SRR34116595 |
|  | Culture | Negative | Negative |
| 51 | Sputum | PRJNA1280714 | SRR34116593 |
|  | Culture | PRJNA1273648 | SRR33884381 |
| 52 | Sputum | PRJNA1280714 | SRR34116592 |
|  | Culture | PRJNA1273648 | SRR33884380 |
| 53 | Sputum | PRJNA1280714 | SRR34116591 |
|  | Culture | PRJNA1273648 | SRR33884379 |
| 54 | Sputum | PRJNA1280714 | SRR34116590 |
|  | Culture | PRJNA1273648 | SRR33884378 |
| 55 | Sputum | PRJNA1280714 | SRR34116589 |
|  | Culture | PRJDB12894 | DRR629209 |
| 56 | Sputum | PRJNA1280714 | SRR34116588 |
|  | Culture | PRJNA1273648 | SRR33884414 |
| 57 | Sputum | PRJNA1280714 | SRR34116587 |
|  | Culture | Negative | Negative |
| 58 | Sputum | PRJNA1280714 | SRR34116586 |
|  | Culture | Negative | Negative |
| 59 | Sputum | PRJNA1280714 | SRR34116585 |
|  | Culture | Negative | Negative |
| 60 | Sputum | PRJNA1280714 | SRR34116584 |
|  | Culture | Negative | Negative |
| 61 | Sputum | PRJNA1280714 | SRR34116582 |
|  | Culture | PRJNA1273648 | SRR33884413 |
| 62 | Sputum | PRJNA1280714 | SRR34116581 |
|  | Culture | PRJDB12894 | DRR629212 |
| 63 | Sputum | PRJNA1280714 | SRR34116580 |
|  | Culture | Negative | Negative |
| 64 | Sputum | PRJNA1280714 | SRR34116579 |
|  | Culture | Negative | Negative |
| 65 | Sputum | PRJNA1280714 | SRR34116578 |
|  | Culture | Negative | Negative |
| 66 | Sputum | PRJNA1280714 | SRR34116577 |
|  | Culture | PRJDB12894 | DRR629214 |
| 67 | Sputum | PRJNA1280714 | SRR34116576 |
|  | Culture | PRJNA1273648 | SRR33884412 |
| 68 | Sputum | PRJNA1280714 | SRR34116575 |
|  | Culture | PRJDB12894 | DRR629218 |
| 69 | Sputum | PRJNA1280714 | SRR34116574 |
|  | Culture | PRJDB19189 | DRR629223 |
| 70 | Sputum | PRJNA1280714 | SRR34116573 |
|  | Culture | PRJNA1273648 | SRR33884411 |
| 71 | Sputum | PRJNA1280714 | SRR34116571 |
|  | Culture | PRJDB19189 | DRR629231 |
| 72 | Sputum | PRJNA1280714 | SRR34116570 |
|  | Culture | Negative | Negative |
| 73 | Sputum | PRJNA1280714 | SRR34116569 |
|  | Culture | Negative | Negative |
| 74 | Sputum | PRJNA1280714 | SRR34116568 |
|  | Culture | PRJDB12894 | DRR629234 |
| 75 | Sputum | PRJNA1280714 | SRR34116567 |
|  | Culture | PRJDB12894 | DRR629197 |
| 76 | Sputum | PRJNA1280714 | SRR34116566 |
|  | Culture | PRJDB12894 | DRR629236 |
| 77 | Sputum | PRJNA1280714 | SRR34116565 |
|  | Culture | PRJDB12894 | DRR629238 |
| 78 | Sputum | PRJNA1280714 | SRR34116500 |
|  | Culture | PRJDB19189 | DRR629198 |
| 79 | Sputum | PRJNA1280714 | SRR34116499 |
|  | Culture | PRJDB12894 | DRR629242 |
| 80 | Sputum | PRJNA1280714 | SRR34116498 |
|  | Culture | PRJDB12894 | DRR629243 |
| 81 | Sputum | PRJNA1280714 | SRR34116496 |
|  | Culture | Negative | Negative |
| 82 | Sputum | PRJNA1280714 | SRR34116495 |
|  | Culture | Negative | Negative |
| 83 | Sputum | PRJNA1280714 | SRR34116494 |
|  | Culture | PRJNA1273648 | SRR33884410 |
| 84 | Sputum | PRJNA1280714 | SRR34116493 |
|  | Culture | Negative | Negative |
| 85 | Sputum | PRJNA1280714 | SRR34116492 |
|  | Culture | PRJNA1273648 | SRR33884409 |
| 86 | Sputum | PRJNA1280714 | SRR34116491 |
|  | Culture | PRJNA1273648 | SRR34136513 |
| 87 | Sputum | PRJNA1280714 | SRR34116490 |
|  | Culture | Negative | Negative |
| 88 | Sputum | PRJNA1280714 | SRR34116489 |
|  | Culture | PRJDB12894 | DRR629249 |
| 89 | Sputum | PRJNA1280714 | SRR34116488 |
|  | Culture | PRJNA1273648 | SRR33884408 |
| 90 | Sputum | PRJNA1280714 | SRR34116487 |
|  | Culture | PRJNA1273648 | SRR33884407 |
| 91 | Sputum | PRJNA1280714 | SRR34116485 |
|  | Culture | PRJNA1273648 | SRR33884406 |
| 92 | Sputum | PRJNA1280714 | SRR34116484 |
|  | Culture | PRJNA1273648 | SRR33884405 |
| 93 | Sputum | PRJNA1280714 | SRR34116483 |
|  | Culture | PRJNA1273648 | SRR33884403 |
| 94 | Sputum | PRJNA1280714 | SRR34116482 |
|  | Culture | PRJNA1273648 | SRR33884402 |
| 95 | Sputum | PRJNA1280714 | SRR34116481 |
|  | Culture | PRJDB19189 | DRR629262 |
| 96 | Sputum | PRJNA1280714 | SRR34116480 |
|  | Culture | PRJNA1273648 | SRR33884401 |
| 97 | Sputum | PRJNA1280714 | SRR34116479 |
|  | Culture | PRJNA1273648 | SRR33884400 |
| 98 | Sputum | PRJNA1280714 | SRR34116478 |
|  | Culture | PRJDB19189 | DRR629204 |
| 99 | Sputum | PRJNA1280714 | SRR34116477 |
|  | Culture | PRJNA1273648 | SRR33884399 |
| 100 | Sputum | PRJNA1280714 | SRR34116476 |
|  | Culture | PRJNA1273648 | SRR33884398 |
| 101 | Sputum | PRJNA1280714 | SRR34116597 |
|  | Culture | PRJNA1273648 | SRR33884397 |
| 102 | Sputum | PRJNA1280714 | SRR34116564 |
|  | Culture | PRJNA1273648 | SRR33884396 |
| 103 | Sputum | PRJNA1280714 | SRR34116563 |
|  | Culture | PRJNA1273648 | SRR33884395 |
| 104 | Sputum | PRJNA1280714 | SRR34116562 |
|  | Culture | PRJNA1273648 | SRR33884394 |
| 105 | Sputum | PRJNA1280714 | SRR34116561 |
|  | Culture | PRJNA1273648 | SRR33884392 |
| 106 | Sputum | PRJNA1280714 | SRR34116560 |
|  | Culture | PRJNA1273648 | SRR34136512 |
| 107 | Sputum | PRJNA1280714 | SRR34116559 |
|  | Culture | PRJNA1273648 | SRR33884391 |
| 108 | Sputum | PRJNA1280714 | SRR34116558 |
|  | Culture | PRJNA1273648 | SRR33884390 |
| 109 | Sputum | PRJNA1280714 | SRR34116557 |
|  | Culture | PRJDB19189 | DRR629221 |
| 110 | Sputum | PRJNA1280714 | SRR34116556 |
|  | Culture | PRJNA1273648 | SRR33884389 |
| 111 | Sputum | PRJNA1280714 | SRR34116554 |
|  | Culture | PRJNA1273648 | SRR33884388 |
| 112 | Sputum | PRJNA1280714 | SRR34116553 |
|  | Culture | PRJNA1273648 | SRR33884387 |

This table provides the correspondence between the sample IDs used in this study and their specific BioProject and Run accession numbers deposited in the public repositories.
